# Imaging Patterns of Recurrent Infarction in the Mechanisms of Early Recurrence in Intracranial Atherosclerotic Disease (MyRIAD) Study

**DOI:** 10.3389/fneur.2020.615094

**Published:** 2021-01-21

**Authors:** Rajbeer S. Sangha, Shyam Prabhakaran, Edward Feldmann, Tristan Honda, Azhar Nizam, George A. Cotsonis, Iszet Campo-Bustillo, Jose G. Romano, David S. Liebeskind

**Affiliations:** ^1^Department of Neurology, University of Alabama at Birmingham, Birmingham, AL, United States; ^2^Department of Neurology, The University of Chicago, Chicago, IL, United States; ^3^Department of Neurology, The University of Massachusetts Medical School-Baystate, Springfield, MA, United States; ^4^Department of Neurology, University of California at Los Angeles, Los Angeles, CA, United States; ^5^Department of Biostatistics and Bioinformatics, Rollins School of Public Health, Emory University, Atlanta, GA, United States; ^6^Department of Neurology, University of Miami, Miami, FL, United States

**Keywords:** ischemic stroke, intracranial atherosclerosis, stroke mechanisms, recurrent infarction pattern, infarct size

## Abstract

**Introduction:** While much is known about recurrent clinical events in patients with intracranial atherosclerotic disease (ICAD), there is limited data on characteristics of recurrent infarcts.

**Methods:** The NIH-funded MyRIAD prospective, observational study was designed to identify mechanisms of ischemia and predictors of recurrence in ICAD. Recurrent infarction was assessed on MRI at 6–8 weeks. We reviewed the DWI/ADC and FLAIR sequences in patients with recurrent stroke and characterized the number of infarcts, infarct location, size, and patterns based on whether they were borderzone (BZ), perforator (SC/P), cortical or territorial (C/T), and mixed. Temporal characteristics were delineated by ADC/FLAIR correlation.

**Results:** Of the 89 patients with 6–8 weeks MRI, 22 (24.7%) had recurrent infarcts in the territory of the symptomatic artery. Recurrent infarcts were evident on DWI in 63.6% and single infarcts in 54.5%. The median recurrent infarct volume was 2.0 cm^3^ compared to median index infarct volumes of 2.5 cm^3^. A mixed infarct pattern was most common (40.9%), followed by borderzone (22.7%), cortical or territorial (27.3%), while only 9.1% were in a perforator artery distribution. Amongst those with a mixed pattern, 8/9 had a borderzone distribution infarct as part of their mixed infarct pattern.

**Conclusion:** These findings provide novel data on the characteristics of early recurrent infarcts in patients with symptomatic ICAD.

## Introduction

Intracranial atherosclerotic disease (ICAD) is the most common cause of ischemic stroke globally and carries the highest risk of stroke recurrence with an estimated 1 year rate of ~12% despite aggressive medical therapy (1–3). The Mechanisms of Early Recurrence in Intracranial Atherosclerotic Disease (MyRIAD) Study (NIH/NINDS) aimed to determine the mechanisms of ischemic stroke in ICAD using imaging biomarkers and determine the rate of early recurrent infarction (4).

Recurrent infarct characteristics may provide valuable information regarding the mechanisms of ischemic recurrence. Previous studies have investigated the correlations between mechanisms of ischemic stroke and infarct patterns on brain imaging in patients with ICAD (5–7). Analysis of infarct patterns in patients with ICAD have demonstrated a correlation between borderzone infarcts and poor distal perfusion (8). In this secondary analysis of MyRIAD, we aimed to describe recurrent infarct characteristics patients enrolled in MyRIAD.

## Methods

### Study Design

The MyRIAD study design has been previously described (4). Eligible patients had a recent (<21 days) ischemic stroke or a transient ischemic attack (TIA) caused by ICAD of the intracranial carotid artery, middle cerebral artery M1 segment, basilar artery, or vertebral artery V4 segment, with 50–99% stenosis, in the absence of proximal cervical arterial stenosis >50% or a cardioembolic source. Ischemic stroke required symptoms lasting >24 h with imaging confirmation of an infarct; TIA had either diffusion weighted imaging (DWI) abnormality or two or more stereotypical events with unequivocally ischemic symptoms. The degree of stenosis was calculated by established methods (9) on digital subtraction angiography (*n* = 23), CT angiography (*n* = 69) or MR angiography (*n* = 26); a flow gap on MR angiography was considered eligible. Eligibility for vascular imaging was reviewed centrally. Eligible patients were >30 years of age, but those of age 30–49 years had either established atherosclerosis in another vascular bed or 2 or more vascular risk factors, and signed informed consent. We excluded those with contraindications to MRI, MR contrast agents, including allergy, creatinine >1.5 mg/dL or GFR <30 mL/min/1.73 m^2^, and those with planned endovascular treatment for ICAD. All enrolled patients were treated with aggressive medical therapy based on the Stenting and Aggressive Medical Management for Preventing Recurrent Stroke in Intracranial Stenosis (SAMMPRIS) trial medical regimen (10). The primary outcome in the MyRIAD study was to assess the incidence of ischemic stroke in the territory of the stenotic artery; secondary outcomes included recurrent TIA and new infarct on MRI at 6–8 weeks.

### Clinical and Imaging Data

We recorded demographic and clinical characteristics, medications, laboratory tests at the time of the index event, and collected all eligibility brain parenchymal and vascular imaging. A brain MRI with FLAIR and DWI/ADC sequences was obtained at 6–8 weeks. Clinical follow-up occurred at 6–8 weeks, 3 months + 15 days, 6 months + 15 days, and 12 months + 21 days to determine if an endpoint had occurred and to record treatment adherence.

### Infarct Patterns and Imaging Characteristics of Recurrent Infarcts

**Figure 1:**
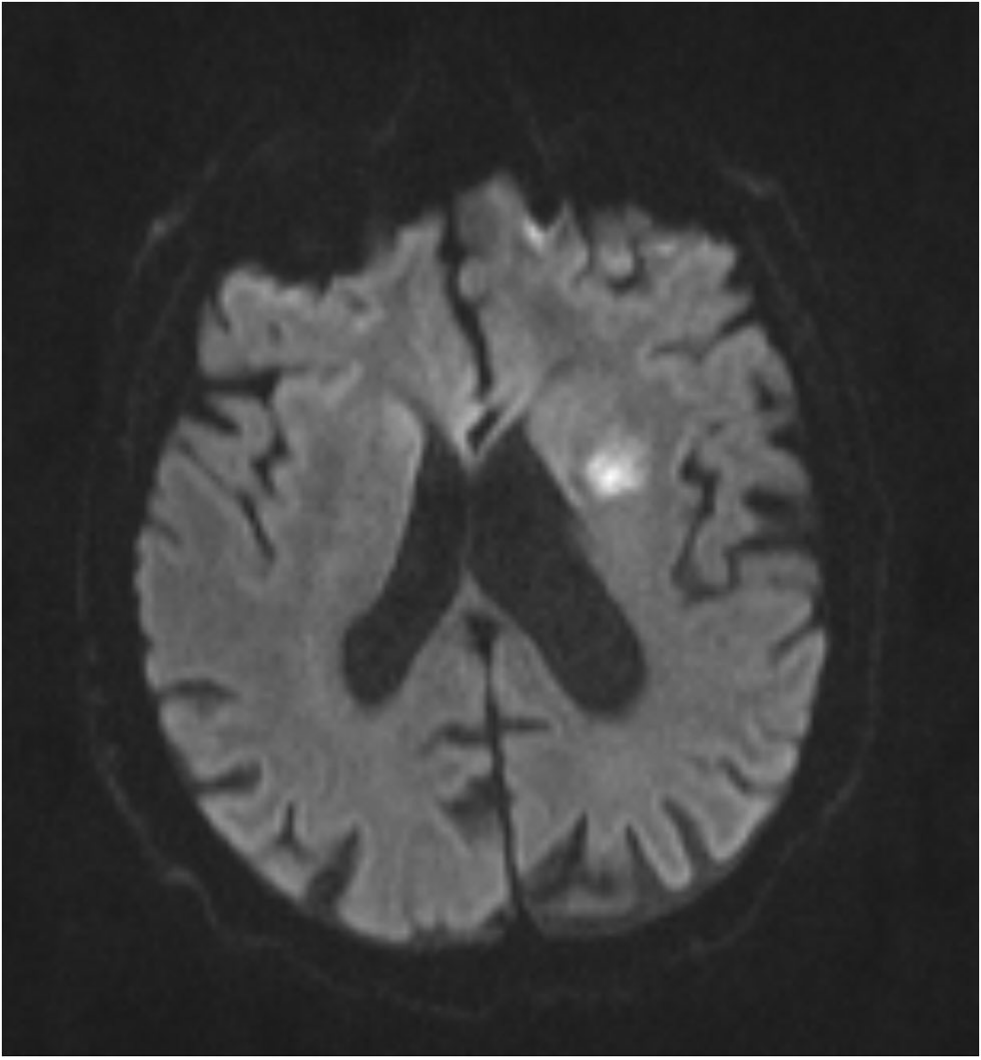
MRI DWI sequence of Perforator Infarct.

**Figure 2:**
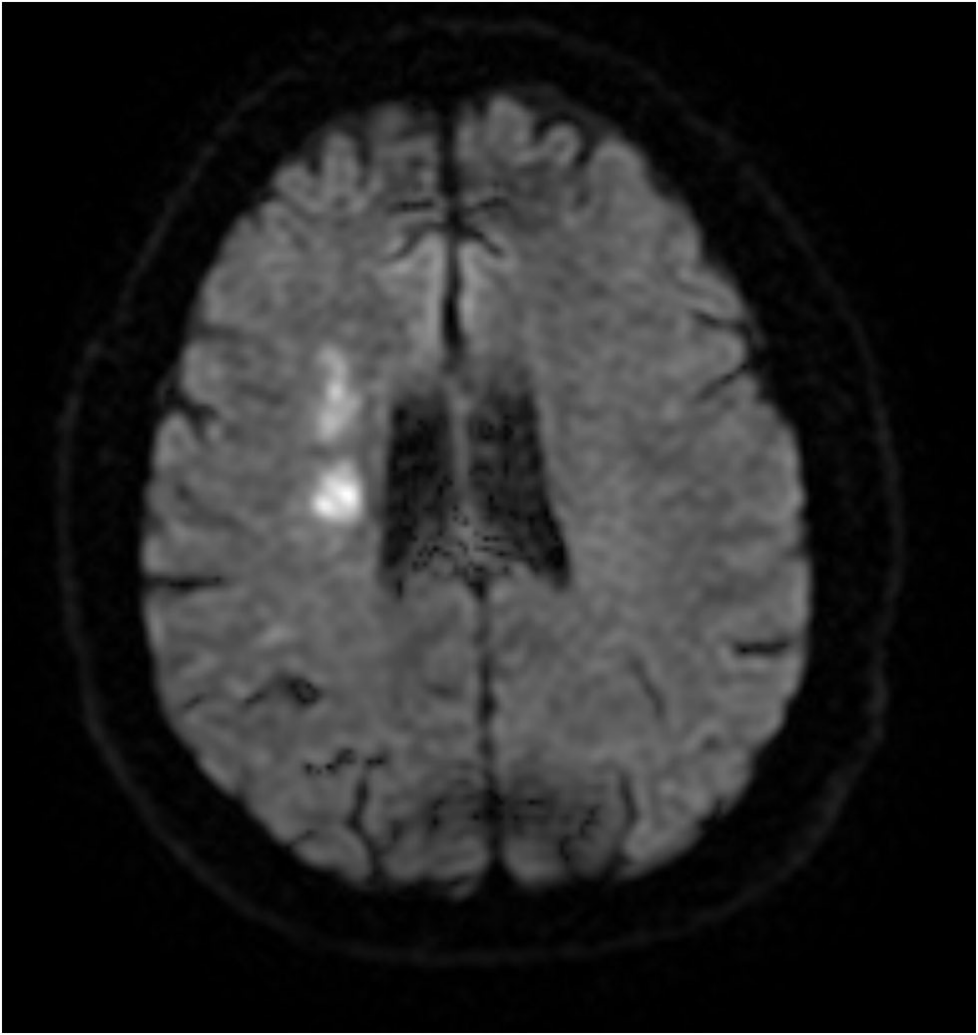
MRI DWI sequence of Borderzone Infarct.

**Figure 3:**
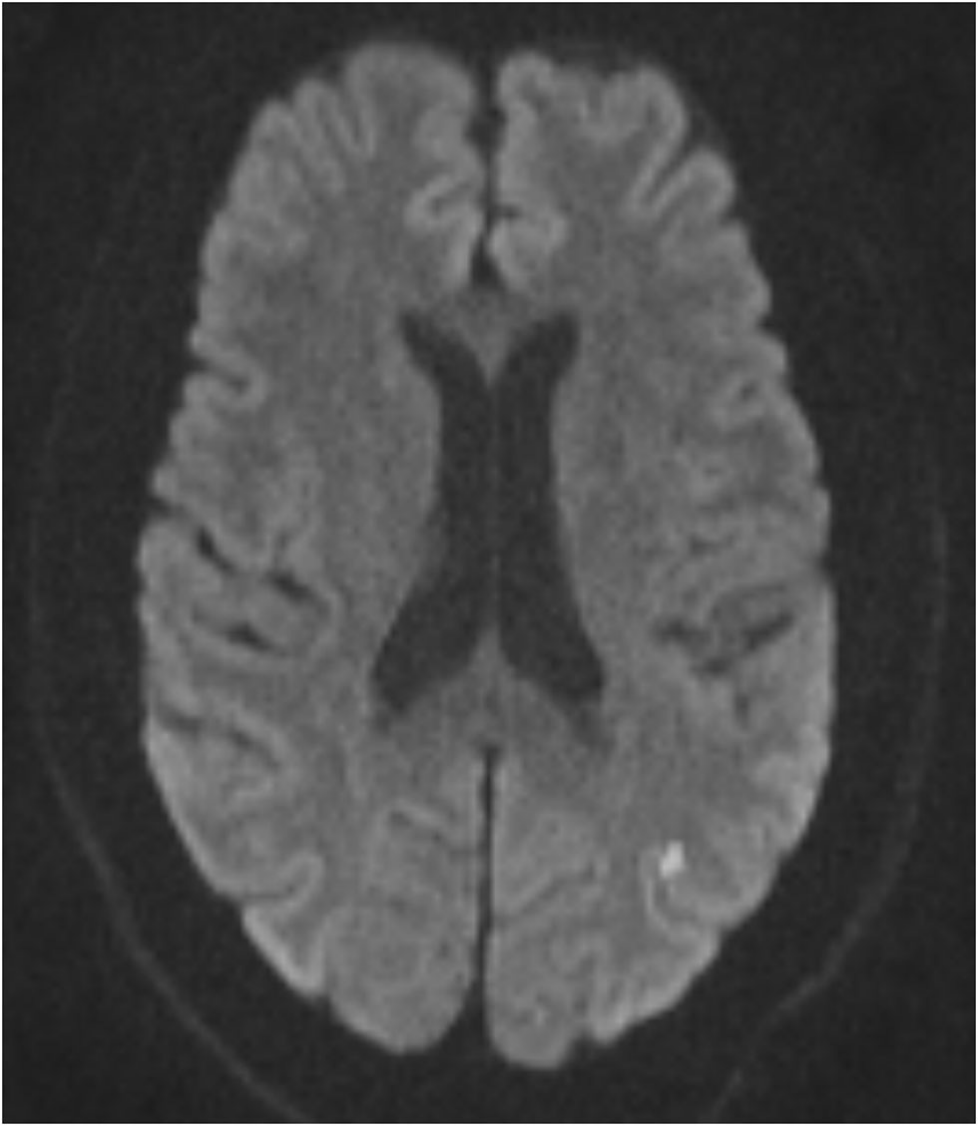
MRI DWI sequence of Cortical/Territorial Infarct.

**Figure 4:**
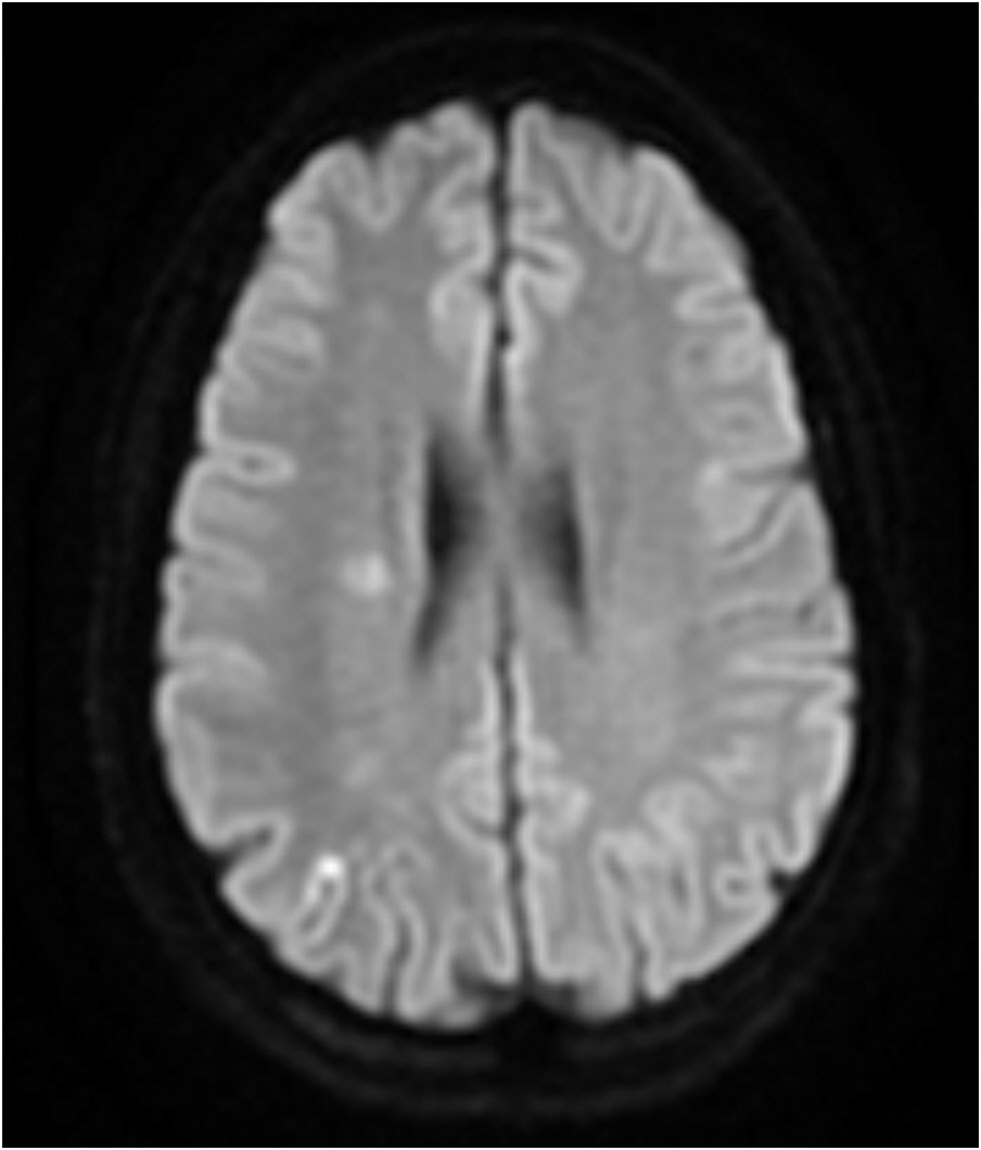
MRI DWI sequence of Mixed Infarct.

The baseline study imaging was evaluated by central readers. Patterns of recurrent infarcts were analyzed by reviewing the MRI FLAIR and DWI sequences by two central adjudicators (R.S.S and D.L). We classified recurrent infarct patterns using a methodology used prior studies of ICAD (5) as follows: (1) Perforator (Figure 1)—single subcortical lesions that result from perforating vessels originating at the site of the stenosis; (2) Borderzone (Figure 2)—lesions that occur in the corona radiata or centrum semiovale and or between cortical borderzone territories, such as between the middle cerebral artery and anterior cerebral artery or the middle cerebral artery and the posterior cerebral artery; (3) Cortical/Territorial (Figure 3)—lesions that occur distal to the region of stenosis but are located in the region supplied by the corresponding intracranial artery; and (4) Mixed (Figure 4)—a combination of cortical and subcortical lesions.

Recurrent infarcts were considered when new DWI/ADC or FLAIR sequence lesion developed compared to the baseline MRI ascertained by 2 independent experienced vascular neurologists (EF, RSS) who reviewed clinical data and imaging studies, including that obtained at baseline, 6–8 weeks, and at the time of a clinical endpoint. Recurrent infarcts were also analyzed for volume of infarcts by comparing the original volume of infarct measured on FLAIR (cm^3^) as well as the volume of FLAIR and DWI lesions at 6–8 weeks MRI or clinical endpoint MRI. Oleasphere 3.0 was used to co-register the baseline and 6–8 weeks MRI FLAIR sequences. Volumes of new infarction were calculated from the subtraction maps after visual verification to remove any artifacts.

Continuous measurements are summarized as mean ± standard deviation, or median ± interquartile range for attributes with skewed distributions.

MyRIAD is supported through a grant by the NIH/NINDS (R01 NS084288). The institutional review board/ethics committee at each participating institution approved this study, which is registered at ClinicalTrials.gov (NCT02121028).

## Results

Of 89 patients with 6–8 weeks MRI, 22 (24.7%) had recurrent infarcts in the territory of the symptomatic artery. The mean age was 57.7 ± 12.2 (mean, SD) years with 14/22 (64%) male, 13/22 (59%) White, 8/22 (36%) African American, 1/22 (5%) Asian, and 4/22 (18%) Hispanic. The location of stenosis was the middle cerebral artery in 14 (63.6%), intracranial carotid artery in 4 (18.2%) and basilar artery in 3 (13.6%). The mean degree of stenosis was 83% (±9%) in 21 cases while 1 had a flow gap on MRA.

The recurrent infarct was evident on DWI in 14 of the 22 (63.6%) patients while in 8 of 22 (36.4%) they were apparent only on FLAIR. Only 5/22 (22.7%) patients had symptomatic presentations in this time period. Recurrent ischemic lesions were single in 12/22 (54.5%) and multiple in 10/22 (45.5%) patients. The median volume of infarcted tissue was 2.0 cm^3^ with interquartile range 5.3 cm^3^ (minimum and maximum volumes 0 and 25.2 cm^3^, respectively). Index infarct volumes were median 2.5 cm^3^ with interquartile range 7 cm^3^ (minimum and maximum 0 and 30.3 cm^3^).

The infarct patterns are exemplified in Table 1. We found a mixed pattern in 9/22 (40.9%), borderzone distribution in 5 (22.7%), cortical or territorial in 6 (27.3%), while only 2 (9.1%) were in a perforator artery distribution. Amongst those with a mixed pattern, 8/9 had a borderzone distribution infarct as part of their mixed infarct pattern.

**Table 1:** Characteristics of patients with recurrent infarct in the MyRIAD cohort.

**Age (years)**	**Sex**	**Eligible event**	**Target vessel**	**Stenosis (%)**	**Recurrent infarct pattern**
48.2	Female	Stroke	LMCA	95	Cortical/Territorial
81.2	Female	Stroke	LMCA	85	Borderzone
35.8	Male	Stroke	RMCA	100	Mixed (B/P)
66.3	Male	Stroke	RMCA	73	Mixed (B/P)
71.9	Female	Stroke	RMCA	89	Mixed (B/P)
65.7	Male	Stroke	LMCA	69	Borderzone
72.1	Male	Stroke	RICA	Flow Gap	Cortical/Territorial
45.3	Female	Stroke	RICA	77	Borderzone
77.1	Male	Stroke	LMCA	99	Perforator
54.7	Male	Stroke preceding TIA	LMCA	84	Cortical/Territorial
54.0	Male	Stroke	LMCA	78	Mixed (B/C)
37.7	Male	Stroke followed by TIA	LMCA	85	Mixed (B/C)
46.8	Female	Stroke preceding TIA	LICA	80	Mixed (B/P)
55.7	Male	Stroke preceding TIA	Basilar	86	Cortical/Territorial
61.2	Male	Stroke	RMCA	94	Borderzone
58.8	Male	Stroke followed by TIA	Basilar	82	Cortical/Territorial
58.0	Female	Stroke	RMCA	79	Mixed (B/C)
52.1	Male	Stroke preceding TIA	RMCA	75	Perforator
44.4	Female	Stroke	RICA	82	Mixed (B/C)
70.2	Male	Stroke	LICA	70	Mixed (P/C)
58.1	Male	Stroke	LMCA	71	Borderzone
54.4	Female	TIA	Basilar	83	Cortical/Territorial

## Discussion

In this secondary analysis of the MyRIAD study, we describe characteristics of recurrent infarcts, with interesting observations. First, patients were noted to have recurrent infarcts that were similar in size to the original index event. Both the index event and recurrent events had lesion volumes of 2–2.5 cm^3^. These data suggest that that a quarter of patients with ICAD will accrue infarcts within 8 weeks with similar volumetric impact on the brain as the index infarct. These cumulative effects of overt and covert infarcts may lead to long-term cognitive and other neurologic consequences and requires further study. Previous studies have shown that infarct size is a predictor of poor outcome in ischemic stroke patients (11). It should be noted that while infarct size and outcome has been reported in lacunar strokes (12), this is the first study to report the size of recurrent infarcts in patients with ICAD. Further studies should focus on determination of patient functional and cognitive outcomes with temporal relation to changes in infarct size.

Second, nearly two thirds of recurrent infarcts had restricted diffusion, indicating that the infarcts were acute and not merely evolution and extensions of the original infarcts. A prior study suggested that while early lesion recurrence may be secondary to a progression of the initial ischemic event, delayed occurrence is a more accurate reflection of a recurrent ischemic event (13). Given that only five of the 22 cases had a clinical stroke recurrence, MRI-defined ischemic lesion recurrence could be a more sensitive and objective marker for stroke recurrence and should be considered in future clinical trials of ICAD (13). It is possible that earlier small recurrent infarcts within areas of baseline FLAIR/T2 signal abnormality could go undetected at the time frame of repeat imaging as the DWI signal abnormality may have normalized. The time course of early infarction recurrence should be evaluated in future studies with earlier repeat imaging. Furthermore, it is difficult to ascertain the effects of silent ischemic lesions (SIL) without clinical manifestations on patient outcomes; further analysis is required to assess changes in patient cognition, physical phenotypes, and quality of life to fully understand whether SIL's will require further changes in medical therapy or medical approaches for patients with symptomatic ICAD.

Finally, the majority of patients with recurrent infarcts had had a borderzone distribution infarct, either alone or in combination with other patterns, at the index event. This suggests that hypoperfusion is a significant mechanism for recurrent events. Similar patterns have been seen in previous analysis of ICAD patients and recurrent patterns of infarcts (5). Patients with a perforator pattern of infarct were less common. This is different from other studies (5) and may be explained by the small sample size.

There are limitations to this study. With a small sample size, it is difficult to draw definitive conclusions regarding the mechanisms of recurrent infarcts. This analysis was also completed *post-hoc* with attendant biases. This study also has some important strengths, including volumetric analysis of recurrent infarct volume and careful ascertainment of infarct recurrence in a meticulously analyzed ICAD cohort.

## Data Availability Statement

The original contributions presented in the study are included in the article/supplementary materials, further inquiries can be directed to the corresponding author/s.

## Ethics Statement

The studies involving human participants were reviewed and approved by the institutional ethics review board at each participating institution approved to participate in MyRIAD. The patients/participants provided their written informed consent to participate in this study.

## Author Contributions

RS, SP, JR, and DL: formulation of research question, data collection, data review, writing manuscript, and editing of manuscript. EF, TH, GC, and IC-B: data collection. AN: statistical analysis, data collection, and editing of manuscript. All authors contributed to the article and approved the submitted version.

## Conflict of Interest

The authors declare that the research was conducted in the absence of any commercial or financial relationships that could be construed as a potential conflict of interest.
